# Rectal cavernous hemangioma: is endoscopic submucosal dissection the new standard of care?

**DOI:** 10.1055/a-2340-8794

**Published:** 2024-06-25

**Authors:** Francesco Cocomazzi, Marco Gentile, Lucia Dimitri, Antonio Capogreco, Roberta Maselli, Alessandro Repici, Francesco Perri

**Affiliations:** 1 Division of Gastroenterology and Endoscopy, “Casa Sollievo della Sofferenza” Hospital, IRCCS, San Giovanni Rotondo, Italy; 2 577188 Pathology Unit, “Casa Sollievo della Sofferenza” Hospital, IRCCS, San Giovanni Rotondo, Italy; 3 Endoscopy Unit, Humanitas Clinical and Research Center – IRCCS, Rozzano, Italy; 4 437807 Department of Biomedical Sciences, Humanitas University, Milan, Italy

Cavernous hemangioma is a benign vascular tumor whose incidence is very low in the gastrointestinal (GI) tract. Usually, it arises from the submucosal vascular plexus. Rectosigmoid is the most frequent location. Anemia, pain, and rectal bleeding are the main symptoms. Endoscopic diagnosis is far from easy: a bluish polypoid lesion, sometimes pedunculated, with superficial vascular congestion is typical. On endoscopic ultrasound (EUS) it appears heterogeneous with hypoechoic and hyperechoic areas, the latter attributable to calcifications. Biopsies should be avoided, as they can cause massive hemorrhage. Sclerotherapy, embolization, and surgery have been the most considered treatment over the years 1 . Endoscopic mucosal resection and endoscopic full-thickness resection have been described, but the best technique should be endoscopic submucosal dissection (ESD) 2 3 .

After performing colonoscopy for hematochezia, a 49-year-old woman was referred to our center. A subepithelial lesion, approximately 18–20 mm, with a bluish rim and superficial congestion, was found in the rectum ( Fig. 1 ). EUS (Olympus, Tokyo, Japan) showed a submucosal, non-homogeneous, predominantly hypoechoic lesion, with anechoic areas and calcification with an acoustic shadow ( Fig. 2 **a, b** ). Magnetic resonance imaging showed a T2 hyperintense neoplasm ( Fig. 3 **a, b** ). A submucosal vascular tumor was suspected; thus, ESD was scheduled.

**Fig. 1:**
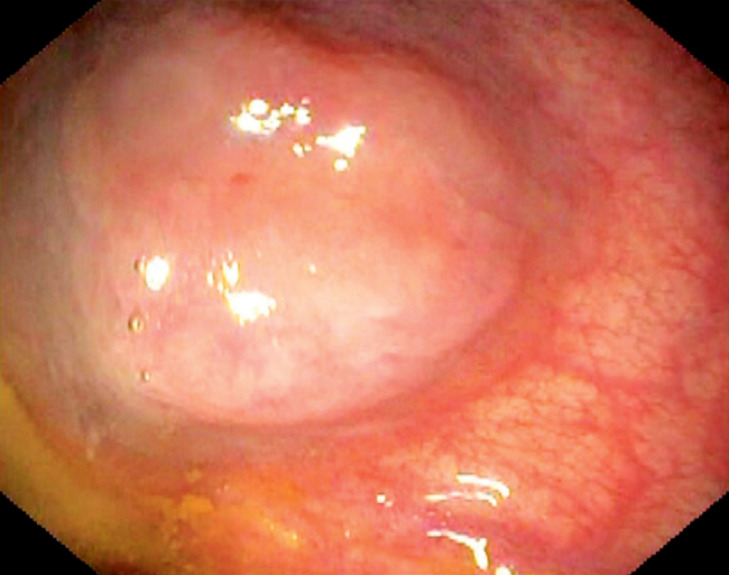
Endoscopic features of the lesion.

**Fig. 2:**
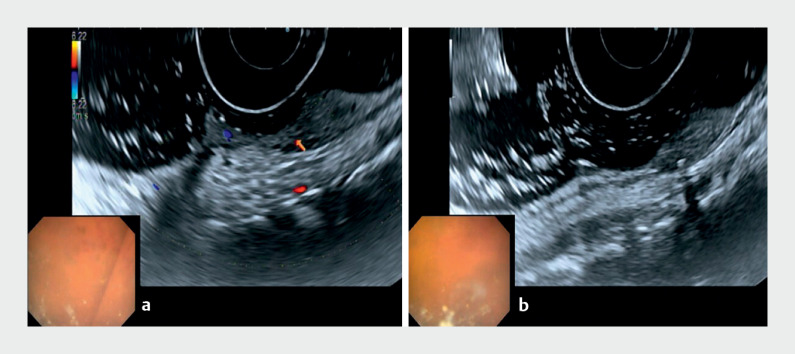
Endoscopic ultrasonographic assessment of the lesion. **a** Submucosal, non-homogeneous, predominantly hypoechoic lesion, with anechoic areas. **b** Calcification with acoustic shadow inside the hemangioma.

**Fig. 3:**
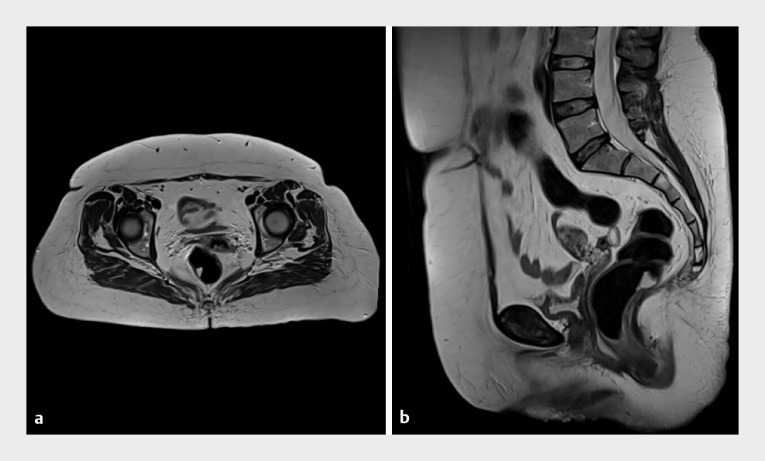
Magnetic resonance appearance of the lesion. **a, b** T2 hyperintense neoplasm in the axial and sagittal plane.

ESD with a HybridKnife (Erbe, Tübingen, Germany) ( Video 1 ) was performed. Given the suspicion of a vascular lesion, the procedure was carried out with great caution to avoid major bleeding. Saline-immersion therapeutic and prophylactic vessel coagulation was performed, as recently described 4 . The patient was discharged asymptomatic the day after. Histological examination confirmed a submucosal cavernous hemangioma with free resection margins ( Fig. 4 **a, b** ).

Endoscopic submucosal dissection of a rectal cavernous hemangioma.Video 1

**Fig. 4:**
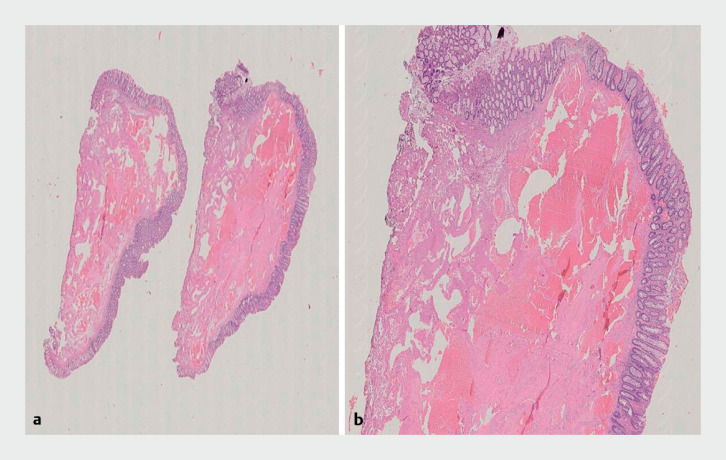
Histological examination of the resected specimen.

This represents the first case of rectal cavernous hemangioma radically removed with ESD, without clip application or antibiotics use and with the adoption of saline-immersion coagulation. As reported 5 , ESD could become the standard of care for these GI lesions, replacing the much more invasive surgery.

Endoscopy_UCTN_Code_TTT_1AQ_2AD_3AD

## Notes

Endoscopy E-Videos https://eref.thieme.de/e-videos
*E-Videos* is an open access online section of the journal *Endoscopy* , reporting on interesting cases and new techniques in gastroenterological endoscopy. All papers include a high-quality video and are published with a Creative Commons CC-BY license. Endoscopy E-Videos qualify for HINARI discounts and waivers and eligibility is automatically checked during the submission process. We grant 100% waivers to articles whose corresponding authors are based in Group A countries and 50% waivers to those who are based in Group B countries as classified by Research4Life (see: https://www.research4life.org/access/eligibility/ ). This section has its own submission website at https://mc.manuscriptcentral.com/e-videos .
